# Prevalence, outcomes and factors associated with adult in hospital cardiac arrests in a low-income country tertiary hospital: a prospective observational study

**DOI:** 10.1186/s12873-015-0047-0

**Published:** 2015-09-16

**Authors:** Davidson Ocen, Sam Kalungi, Joseph Ejoku, Tonny Luggya, Agnes Wabule, Janat Tumukunde, Arthur Kwizera

**Affiliations:** Department of Anaesthesia, Makerere University College of Health Sciences, Mulago National Referral Hospital, Kampala, Uganda; Department of Pathology, Makerere University College of Health Sciences, Mulago National Referral Hospital, Kampala, Uganda

## Abstract

**Background:**

Research on cardiac arrest and cardiopulmonary resuscitation (CPR) has considerably increased in recent decades, and international guidelines for resuscitation have been implemented and have undergone several changes. Very little is known about the prevalence and management of in-hospital cardiac arrest in low-resource settings. We therefore sought to determine the prevalence, outcomes and associated factors of adult inpatients with cardiac arrest at a tertiary referral hospital in a low-income country.

**Methods:**

Upon obtaining institutional approval, we conducted a prospective observational period prevalence study over a 2-month period. We recruited adult inpatients with cardiac arrest in the intensive care unit and emergency wards of Mulago Hospital, Uganda during the study period. We reviewed all files and monitoring charts, and also any postmortem findings. Data were analyzed with Stata 12 and statistical significance was set at *p* < 0.05.

**Results:**

There was a cardiac arrest in 2.3 % (190) of 8,131 hospital admissions; 34.5 % occurred in the intensive care unit, 4.4 % in emergency operating theaters, and 3.0 % in emergency wards. A majority (63.2 %) was unwitnessed, and only 35 patients (18.4 %) received CPR. There was return of spontaneous circulation (ROSC) in 14 (7.4 %) cardiac arrest patients. Survival to 24 h occurred in three ROSC patients, which was only 1.6 % of all cardiac arrest patients during the study period. Trauma was the most common primary diagnosis and HIV infection was the most common co-morbidity.

**Conclusion:**

Our hospital has a high prevalence of cardiac arrest, and low rates of CPR performance, ROSC, and 24-hour survival. Single provider CPR; abnormal temperatures as well as after hours/weekend CAs were associated with lower survival rates.

## Background

According to the Utstein definition, cardiac arrest is a sudden cessation of cardiac mechanical function as evidenced by absence of detectable pulse, absent or gasping breath, and/or loss of consciousness [1]. Since its introduction and recommendation in the 1960s, cardiopulmonary resuscitation (CPR) has become the standard treatment and has been widely used for the management of cardiac arrest. Initial survival after CPR is up to 50 %, but there is low survival to hospital discharge, ranging from 11 to 37 % [1]. Considerable regional variation in the incidence and outcome of cardiac arrest within the United States has been reported [2–10].

Many studies have identified variables associated with a favorable neurological survival after in-hospital cardiac arrest (IHCA): younger age, witnessed arrest, initial cardiac arrest rhythm of ventricular fibrillation or pulseless ventricular tachycardia with a defibrillation time of ≤2 min, baseline neurological status without disability, arrest location in a monitored unit, shorter response time and duration of resuscitation, lack of need for mechanical ventilation after resuscitation, white ethnicity, recent surgery, trauma, presumed cardiac etiology, subjects with an internal cardioverter defibrillator, hypotension prior to the arrest, angiotensin converting enzyme inhibitor treatment, anti-arrhythmic drugs at the time of arrest, and rescuer procedural experience. Pre-arrest prognostic factors of advanced age, unwitnessed arrest, comorbidities, sepsis, cancer, renal failure, black ethnicity, re-arrest, a homebound lifestyle, prolonged duration of CPR, and increasing total dose of adrenaline during resuscitation are significantly associated with poor survival [1, 9–15].

In recent years, research on resuscitation has considerably increased. Guidelines for cardiac resuscitation have been implemented at an international level and have undergone several substantial changes [16]. The institution of cardiac arrest teams for in-hospital resuscitation has become a standard for many medical centers worldwide. However, the reported survival rates vary significantly according to the center, patient and event characteristics [1]. A study performed in South Africa to assess the return of spontaneous circulation (ROSC) in out-of-hospital cardiac arrest patients reported a low rate of ROSC of 18 % in all cases managed by an emergency team and was attributed to a long response time [17]. A pediatric study in Kenya showed that cardiopulmonary arrest after admission had a very poor prognosis, with 16 % surviving initial CPR and none surviving to discharge. Infectious diseases were the main underlying causes of arrest [18].

In this study, we sought to determine the prevalence, outcomes and associated factors of adult in-hospital cardiac arrest in the low-income setting of Uganda.

## Methods

We conducted a period prevalence study over a 2-month period from February to April 2014 in Mulago National Referral and Teaching Hospital, located in Kampala, the capital city of Uganda.

Mulago Hospital has a bed capacity of over 1500 beds and serves a national population of over 33,000,000 people. The hospital has 13 operating theaters with a total of 24 operating tables, and conducts over 8,000 operations per annum. It has 12 general intensive care unit (ICU) beds, with only six functional at the time of the study, and admits 150–250 patients annually [19]. The accident and emergency unit comprises a reasonably well-equipped five-bed trauma unit, a casualty unit, and separate surgical and medical emergency wards. Mulago Hospital has almost all types of medical professionals with some having formal and informal training on Basic Life Support, Advanced Cardiac Life Support (ACLS), and Advanced Trauma Life Support, but it is understaffed. No programmed refresher emergency courses exist in the hospital. There are no established in-hospital medical emergency response systems, cardiac arrest team or management protocols. Emergency kits such as airway assist devices, emergency drugs, oxygen, suction units, monitors and defibrillators are unevenly distributed, although the ICU and operating theaters where anesthesiologists work are better equipped than the general and emergency wards.

This study included all adult inpatients aged 18 years and older that were diagnosed with an index cardiac arrest in the Accident and Emergency Department, ICU and all operating theaters after admission to Mulago Hospital. Patients with out-of-hospital cardiac arrest or IHCA in wards outside the study wards were excluded. For purposes of the study, any patient who was discovered unresponsive with an absent carotid pulse and /or clinically confirmed dead was considered to have suffered cardiac arrest. There were study assistants on 24-hour standby recruited from among ward staff to ensure that no patient was missed.

Patient data were collected using a pretested data collection tool. Permission to conduct the study and waiver of consent was obtained from the research and ethics committee, school of medicine, Makerere university college of health sciences (REC REF 2014/012). Data were collected twice every day (morning 8:00–11:00 and evening 17:00–19:00) in the study wards. Ward/unit reports for cardiac arrest and all deaths for the previous 12–24 h were reviewed along with postmortem reports from the mortuary, if performed. The teams managing the patients made the diagnosis of cardiac arrest. All consequent deaths were considered related to the cardiac arrest. The data collected included the following: patient demographics, any significant comorbidity, provisional diagnosis, pre-arrest vital signs, witnessed or un-witnessed arrest, time of arrest, activation of an emergency response, time of initiation of resuscitation, resuscitation/interventions given, and outcome of resuscitation. If there was survival (ROSC), immediate vital signs, electrolyte level, random blood sugar, post-arrest care, re-arrest, and survival to 24 h were determined. For all deaths, postmortem findings (if an autopsy was performed) were recorded.

The main dependent outcomes were: ROSC following cardiac arrest and patient demographic factors. Clinical factors, hospital factors, and 24-hour survival were considered independent outcomes. Data analysis was performed using Stata 12 (Stata Corp., College Station, TX, USA). Categorical variables are presented as proportions and percentages. Variables considered clinically relevant were entered into the bivariate analysis to determine the association of ROSC and each predictor.

## Results

We recruited a total of 190 cardiac arrest patients from the study wards during the study period. This was out of a total of 8,131 patient admissions (Table 1). There were 136 (71.58 %) males and 54 (28.42 %) females; the majority (66.5 %) were young adults (18–44 years); 83.2 % of cardiac arrests occurred in emergency wards/units; and the duration of illness was less than 1 week in 74.3 %.

**Table 1:** Baseline characteristics

Variable	Distribution (*n* = 190)
	n (%)
Age (years)	
18–44	119 (62.6 %)
45–65	37 (19.5 %)
>65	23 (12.1 %)
Unknown	11(5.8 %)
Sex	
Male	136 (71.6 %)
Female	54 (28.4 %)
Unit/Ward	
ICU	20 (10.5 %)
Emergency wards	158 (83.2 %)
Theater	12 (6.3 %)
Duration of illness in days	
≤ 7	139 (74.3 %)
> 7	48 (25.7 %)
Trauma type	
Head injury	50 (76.9 %)
Multiple trauma	15 (23.1 %)

*ICU* Intensive care unit

The prevalence of death from in-hospital cardiac arrest in the study wards during the study period was 2.3 % (95 % confidence interval (CI): 2.0–3.0 %). The prevalence in the ICU was 34.5 % (95 % CI: 22.5–48.1 %), in operating theaters was 0.4 % (95 % CI: 0.2–0.8 %), and in the emergency wards was 3.0 % (95 % CI: 2.0–2.7 %). Trauma was the most common admission diagnosis (58 %) with 76.9 % of all trauma cases being severe head injuries. Trauma was followed by sepsis, hypertension, central nervous system (CNS) disease, heart disease, diabetes mellitus, and liver disease. Comorbidity was recorded in only 62 patients. HIV infection, hypertension, cancer, diabetes mellitus, kidney, liver and CNS diseases constituted 45, 22, 14, 13, 2, 2, and 2 %, respectively.

The cardiac arrest was un-witnessed in the majority of patients recruited (120/190; 63.2 %). Of the 70 arrests witnessed, two were witnessed by attendants and the rest by medical staff. Cardiac arrests occurred during the day, night and weekend in 36.0, 27.4 and 36.6 %, respectively (Table 2). Only 43 patients (22.6 %) had activation of an emergency response protocol, and of these, only 35 received CPR. Reasons for no CPR in the witnessed cases were not given (no reason) in 32 (91.4 %) and brain death in 3 patients (8.6 %). The response time recorded was 0–2 min in 53.6 %, and more than 2 min in 46.4 %. The average CPR duration was 24.4 min (95 % CI: 11–86 min).

**Table 2:** Event characteristics of participants

Variable	Distribution (*n* = 190)
	n (%)
Witnessed arrest	
No	120 (63.2 %)
Yes	70 (36.8 %)
Who witnessed arrest	
Attendant	2 (2.9 %)
Medical personnel	68 (97.1 %)
Time of arrest	
Day, 8:00–19:59	69 (36.0 %)
Night, 20:00–7.59	52 (27.4 %)
Weekend	69 (36.6 %)
Call for help (activation of emergency system)	
No	27 (38.5 %)
Yes	43 (61.5 %)
CPR given in witnessed cases	
No	35 (50 %)
Yes	35 (50 %)
Reasons for no CPR in the witnessed cases	
No reason	32 (91.4 %)
Considered brain dead	3 (8.6 %)

*CPR* Cardiopulmonary resuscitation

Blood pressure, motor Glasgow Coma Scale score, pulse rate, oxygen saturation, respiratory rates, random blood sugar, and body temperature were recorded in 69.47, 69.47, 56.84, 39.47, 27.89, 16.84, and 12.63 % of the recruited patients, respectively (Table 3). Mean arterial pressure was abnormal/hypotensive (<65 mmHg) in 62.9 %, heart rate was abnormal (either <60 or >120 per minute) in 66.7 %, random blood sugar was abnormal (<3.5 or >11 mmol/L) in 84.4 %, oxygen saturation was less than 94 in 74.7 %, and the Glasgow Coma Scale score was very low in half the patients.

**Table 3:** Recording of pre-arrest vital signs

Variable	Distribution
	n (%)
Mean arterial pressure	
Normal	49 (37.1 %)
Abnormal	83 (82.9 %)
Pulse rate	
Normal	39 (33.3 %)
Abnormal	78 (66.7 %)
Temperature	
Normal	5 (20.8 %)
Abnormal	19 (79.2 %)
Random blood sugar	
Normal	5 (15.6 %)
Abnormal	27 (84.4 %)
SPO_2_	
Normal	19 (25.3 %)
Abnormal	56 (74.7 %)
Glasgow Coma Scale – motor	
1–3	66 (50.0 %)
4–5	37 (28.0 %)
6	29 (22.0 %)

Note: All respiratory rates were normal; however, this was only in 53 participants

Survival after initial CPR (ROSC) occurred in only 14 patients (7.4 %) and survival to 24 h in only three of these patients (25 %) or 1.6 % of the total study cohort (Table 4). Half (50 %) of the post-arrest patients were managed in the ICU, 28.8 % in general wards and 21.4 % in emergency wards (Table 4). A postmortem was performed in 75 patients, and the findings were not considered in the clinical diagnosis in 24 % of these patients (Table 4).

**Table 4:** Outcome of cardiac arrest

Variable	Distribution (*n* = 190)
	n (%)
ROSC	
No	176 (92.6 %)
Yes	14 (7.4 %)
Postmortem	
No	111 (59.7 %)
Yes	75 (40.3 %)
Postmortem included in clinical diagnosis	
No	18 (24.0 %)
Yes	57 (76.0 %)
Post-arrest care admission	
ICU	7 (50.0 %)
General wards	4 (28.6 %)
Emergency wards	3 (21.4 %)
24-hour survival	
No	11 (78.6 %)
Yes	3 (21.4 %)
Any re-arrest	
No	3 (21.4 %)
Yes	11 (78.6 %)

*ROSC* Return of spontaneous circulation

ROSC occurred in 25 % of ICU patients, 2.5 % of emergency ward patients and 45.5 % of operating theater patients. Low rates of ROSC were associated with admission diagnosis of trauma and sepsis; comorbidity of HIV; cardiac arrest occurring in emergency wards (*p* = 0.003; OR: 0.1, 95 % CI: 0.02–0.46) and over the weekend (*p* = 0.042; OR: 0.20, 95 % CI: 0.04–0.09) (Table 5).

**Table 5:** Factors associated with ROSC

Risk factor	Return of spontaneous circulation		
	No *N* = 21 (%)	Yes *N* = 14 (%)	p-value
Age			
18–44	14 (67.5)	7 (50.0)	
> 45	7 (20.5)	7 (28.6)	0.48
Sex			
Male	15 (72.2)	9 (64.3)	
Female	6 (27.8)	5 (35.7)	0.72
Unit/Ward			
ICU &Emergency wards	20 (9.1)	8 (28.6)	
Operating theater	1 (3.4)	6 (42.8)	1.0
Duration of illness in days			
≤ 7	16(74.6)	10 (71.4)	
> 7	5 (25.4)	4 (28.6)	1.0
Trauma type*			
Head injury	17(79.3)	4 (66.7)	
Polytrauma	4 (20.7)	2 (33.3)	0.58
Significant Admission diagnosis			
Trauma	12 (56.2)	7 (87.5)	0.06
Sepsis	9 (23.1)	0	
Comorbidity			
Hypertension & Diabetes	6(20.3)	3(66.7)	
HIV	5 (47.5)	0	0.25
Time of arrest			
8:00–19:59	7 (33.7)	10 (71.4)	
20:00–7.59 and Weekend	14(27.9)	4 (14.3)	0.04
Pre-arrest mean arterial pressure			
Normal	8(37.1)	6 (42.9)	
Abnormal	13 (62.9)	8 (57.1)	1.0
Pre-arrest pulse rate			
Normal	7 (34.3)	4 (28.3)	
Abnormal	14 (65.7)	9 (71.7)	1.0
Pre-arrest temperature			
Normal	4 (19.1)	14 (100)	
Abnormal	17 (80.9)	0	0.01
Pre-arrest random blood sugar			
Normal	4 (19.1)	0	
Abnormal	17(80.9)	14 (100)	0.13
Pre-arrest SPO_2_			
Normal	5 (23.5)	6 (42.9)	
Abnormal	16 (76.5)	8 (57.1)	0.28
Pre-arrest Glasgow Coma Scale			
<6	17 (51.2)	8(57.1)	
>6	4 (21.5)	6 (32.9)	0.15
Any call for help			
No (single provider CPR)	17 (81)	0	
Yes	4 (19)	14 (100)	0.01
Response time			
Immediate (0–2 min)	11 (52.4)	9(64.3)	
Delayed (>2 min)	10 (47..6)	5 (35.7)	0.72

## Discussion

We conducted a prospective observational period prevalence study of IHCA patients to determine the prevalence, patient factors, and effectiveness of the hospital system in managing cardiac arrest. Compared with the literature, our hospital had a high prevalence of IHCA. The prevalence was 30 times higher in emergency wards, 100 times higher in operating theaters and about 10 times higher in general wards than previously reported rates of 1–6 cardiac arrests per 1000 general admissions [4, 20], and 0.5 arrests per 10,000 undergoing anesthesia in operating theaters [21]. We also noted that there was a lack of adequate patient monitoring to identify those at risk of cardiac arrest.

In contrast to most studies [1, 4, 11], the majority of our patients were young adults aged less than 45 years. This reflects Uganda’s population, which is comparatively young. Additionally, the majority of our patients were mainly trauma cases, and, as expected, the majority of trauma patients were young males, many of who drove motorcycles, with the commonest mode of trauma being head injury. Our study was comparable with previous reports of a majority of males (60.4–62.4 %) in cardiac arrest patients in the ICU and operating theaters [20, 22]. The most common associated diagnoses in this study were trauma, sepsis, hypertension, HIV-related CNS infections, and non-communicable disease. At postmortem, 24 % of pathological diagnoses were not considered in the final clinical diagnoses. This was much lower than in a prior study, which found a rate of 45 % in HIV-positive patients and 42 % in HIV-negative patients [23]. This discrepancy may be explained by the high rate of trauma in our study population.

Our study has provided evidence of poor recording and monitoring of vital signs (Table 4) in emergency wards. This may be explained by the absence of monitoring charts in the emergency wards, staff shortages, and a lack of monitoring equipment. We also observed that hypotension; abnormal blood sugar, hypoxia, and low motor Glasgow Coma Scale scores were related to cardiac arrest in our setting. It is notable that these are signs that can allow treatment and prevention of cardiac arrest.

Our study also showed low rates of witnessed arrest and activation of an emergency response, which we attribute to the absence of monitoring equipment, and poor staff-to-patient ratios. However, when an emergency response occurred, it was usually immediate. One limitation of our study is that we were unable to determine the quality of the CPR given. Additionally, we were unable to characterize the arrest by electrical activity, especially considering that pulseless electrical activity or asystole, bradyarrhythmia and ventricular fibrillation or ventricular tachycardia have been suggested as independent factors for ROSC and long-term survival post-cardiac arrest [1, 4].

We also found a very low rate of ROSC and 24-hour survival compared with similar studies [4, 5, 22]. While Trauma and sepsis were the admission diagnosis weakly associated with low ROSC, abnormal temperatures were the clinical parameters significantly associated with low ROSC. This is probably due to the poor nature of pre-hospital care and inadequate resuscitation in our setting. Cardiac arrest occurring after hours (in the night) and over the weekend were associated with lower rates of ROSC. This also applied when there was no call for help. This is probably due to the fact that staffing was thin after hours and as a result CPR was single provider managed. Another weakness in our study was that we did not follow up on survival to discharge.

Only half of those who had ROSC were managed in the ICU, and the rest were managed in general and emergency wards. This is in contrast to the 2010 ACLS guidelines, which recommend that all post-cardiac arrest patients should be managed in an ICU [6]. Our ICU had a functional capacity of only six beds at the time of the study [19], thus limiting admission of post-cardiac arrest patients to the ICU. The dearth of critical care units in low-income settings has been well documented in several studies [19, 24–28], however, the 2 key interventions that have been documented to improve outcomes i.e early recognition of IHCA and prompt initiation of CPR also cost the least to institute [29]. We also noted the lack of arrest protocols in the study wards (apart from the ICU) and a lack of skill among medical workers with regards to detection and arrest management. An additional limitation to the study was the poor documentation of clinical parameters. This contributed to incomplete data that confounded our analysis.

## Conclusion

Our study found a high prevalence of cardiac arrest, and low rates of recognition of cardiac arrest, CPR performance, ROSC, and 24-h survival. Trauma was the most common primary diagnosis. Cardiac arrests occurring at night; over the weekend; in patients with abnormal temperatures and those managed by single provider CPR were associated with particularly low survival. The results of this study may not be applicable to other hospitals in other regions since our study was based on data from a single tertiary teaching hospital in Uganda, but they give an insight into problematic arrest care in a hospital in a low-income country. We believe that a locally implemented, strong in-hospital chain of care, which is customizable to specific requirements, may be the best way to improve outcomes in individual hospitals.

## References

[1] Sandroni C, Nolan J, Cavallaro F, Antonelli M (2007). In-hospital cardiac arrest: incidence, prognosis and possible measures to improve survival. Intensive Care Med.

[2] Cope AR, Quinto DN, Dove AF Sloan JP, Dave SH (1987). Survival from cardiac arrest in the Accident and Emergency Department. J R Soc Med.

[3] Koumenhoven WB, Jude JR, Knickerbocker GG (1960). Closed chest cardiac massage. JAMA.

[4] Saghafina M, Motamedi K, Piyaie M, Rafati H, Saghafi A, Jalali A (2010). Survival after in-hospital cardiopulmonary resuscitation in a major referral center. Saudi J Anaesth.

[5] Chan PS, Nichol G, Kruholz HM, Spertus JA, Jones PG, Peterson ED (2009). Racial differences in survival after in-hospital cardiac arrest. JAMA.

[6] Peberdy MA, Callaway CW, Neumar RW, Geocadin RG, Zimmerman JL, Donnino M (2010). 2010 Anerican Heart AssociationGuidelines for Cardiopulmonary resuscitation and Emergency Cardiovascular care. Circulation..

[7] Haukoos JS, Witt G, Gravitz C, Dean J, Jackson DM, Candlin T (2010). Outcome of out of hospital cardiac arrest in Denver, Colorado^;^ epidemiology and outcome. Acad Emerg Med.

[8] Rosamond W, Flegal K, Furie K, Go A, Greenlund K, Haase N (2008). Heart disease and stroke statistics–2008 update: a report from the American Heart Association Statistics Committee and Stroke Statistics Subcommittee. Circulation.

[9] Nichol G, Thomas E, Callaway CW, Hedges J, Powell JL, Aufderheide TP (2008). Regional variation in out-of-hospital cardiac arrest incidence and outcome. JAMA.

[10] Chan PS, Nichol G, Krumbolz HM, Spertus JA, Nallamothu BK (2009). Hospital variation in time to defibrillation after in-hospital cardiac arrest. Arch Intern Med.

[11] Bonnin MJ, Pepe PE, Clark PS (1993). Survival in the elderly after out-of-hospital cardiac arrest, city of Houston center for resuscitation and emergency medical services. Crit Care Med.

[12] Iwani T, Nichol G, Hiraide A, Hayashi Y, Nishiuchi T, Kajino K (2009). Continuous improvement in the chain of survival/increased survival after out-of-hospital cardiac arrests, a large scale population based study. Circulation.

[13] Ehlenbach WJ, Barnato AE, Curtis IR, Kreuter W, Koepsell TD, Deyo RA (2009). Epidemiological study of in-hospital cardiopulmonary resuscitation in the elderly. New Eng J Med.

[14] Nadkarni VM, Larkin GL, Peberly MA, Carey SM, Kaye W, Mancini ME (2006). The first documented rhythm and the clinical outcome from in-hospital cardiac arrest among children and adults. JAMA.

[15] Mönhle P, Huge V, Jan P, Weig I, Atzinger R, Kreimeier U (2012). Survival after cardiac arrest and changing the task profile of the cardiac arrest team in the tertiary center. Scientific World J.

[16] Nolan J (2005). European resuscitation council guidelines for resuscitation 2005:section1, introduction. Resuscitation.

[17] Stein C (2009). Out of hospital cardiac arrest cases in Johannesburg, South Africa; a first glimpse of short term outcome from a paramedic clinical learning database. Emerg Med J.

[18] Olotu A, Ndiritu M, Ismael M, Mohammed S, Mithwani S, Maitland K (2009). Characteristics and outcome of cardiopulmonary resuscitation in hospitalized African children. Resuscitation.

[19] Kwizera A, Dünser M, Nakibuuka J (2012). National intensive care unit bed capacity and ICU patient characteristics in a low income country. BMC Res Notes.

[20] Chakravarthy M, Mitra S, Nonis L (2012). Outcomes of in-hospital, out of intensive care and operation theatre cardiac arrests in a tertiary referral hospital. Indian Heart J.

[21] Kubota Y, Toyoda Y, Kubota H, Ueda Y, Asada A, Okamoto T (1994). Frequency of anesthetic cardiac arrest and death in the operating room at a single general hospital over a 30-year period. J Clin Anesth.

[22] Kutsogiannis DJ, Bagshaw SM, Laing B, Brindley PG (2011). Predictors of survival after cardiac or respiratory arrest in critical care units. CMAJ.

[23] Cox JA, Lukande RL, Nelson AM (2012). An autopsy study describing causes of death and comparing clinico-pathological findings among hospitalized patients in Kampala, Uganda. Acad J.

[24] Vincent J-L, Marshall JC, Ñamendys-Silva SA, François B, Martin-Loeches I, Lipman J (2014). Assessment of the worldwide burden of critical illness: the Intensive Care Over Nations (ICON) audit. The Lancet Respiratory Medicine.

[25] Firth P, Ttendo S (2012). Intensive care in low-income countries—a critical need. New England Journal of Medicine.

[26] Baker T, Lugazia E, Eriksen J, Mwafongo V, Irestedt L, Konrad D (2013). Emergency and critical care services in Tanzania: a survey of ten hospitals. BMC Health Services Research.

[27] Bainbridge D, Martin J, Arango M, Cheng D (2012). Perioperative and anaesthetic related mortality in developed and developing countries: a systematic review and metaanalysis. The Lancet.

[28] Adhikari NK (2013). Patient safety without borders: measuring the global burden of adverse events. BMJ Qual saf.

[29] Murugiah K, Chen SI, Dharmarajan K, Nuti SV, Wayda B, Shojaee A (2014). Most Important Outcomes Research Papers on Cardiac Arrest and Cardiopulmonary Resuscitation. Circ Cardiovasc Qual Outcomes.

